# Supplementary material for: Enhancing chemotherapy response prediction via matched colorectal tumor-organoid gene expression analysis and network-based biomarker selection
Source: Transl Oncol. 2025 Jan 3;52:102238. doi: 10.1016/j.tranon.2024.102238 (PMC11754497; doi:10.1016/j.tranon.2024.102238)
Supplement: Supplementary file 1 [file mmc1.docx]

**Supplementary Figure 1 - Showcases the cross-validation (CV) errors and ROC curves of the Ridge, RF, and ensemble models.** On the top left are boxplots of 100 repeated CV errors. The t-test results between Ridge vs ensemble and RF vs ensemble are displayed at the top of the boxplots, where "**" represents P-values < 0.01 and "****" signifies P-values < 0.0001. On the top right are the ROC curves from testing on the binary label of the OS results of GSE171680 across the three models. The area under the curve (AUC) values are labeled for each model. The bottom shows the KM survival curves and log-rank tests for each model. All results indicate that the ensemble model consistently outperforms the other models, providing the most optimal outcomes.

**Supplementary Figure 2 - Drug-response predictions for 5-FU-based treated samples of six independent datasets with 35 hub genes selected from the consensus WGCNA**

**Supplementary Figure 3 - Drug-response predictions for 5-FU-based treated samples of six independent datasets with candidate genes selected from WGCNA Model 1**

**Supplementary Figure 4 - Drug-response predictions for 5-FU-based treated samples of six independent datasets with candidate genes selected from WGCNA Model 2**

**Supplementary Figure 5 - Drug-response predictions for 5-FU-based treated samples of six independent datasets with candidate genes selected from WGCNA Model 3**

**Supplementary Figure 6 - Drug-response predictions for 5-FU-based treated samples of six independent datasets with candidate genes selected from filtering criterion 1 of gene association tests**

**Supplementary Figure 7 - Drug-response predictions for 5-FU-based treated samples of six independent datasets with candidate genes selected from filtering criterion 2 of gene association tests**
