# Supplementary material for: Enhancing chemotherapy response prediction via matched colorectal tumor-organoid gene expression analysis and network-based biomarker selection
Source: Transl Oncol. 2025 Jan 3;52:102238. doi: 10.1016/j.tranon.2024.102238 (PMC11754497; doi:10.1016/j.tranon.2024.102238)
Supplement: Supplementary file 2 [file mmc2.pdf]

Fig. S1

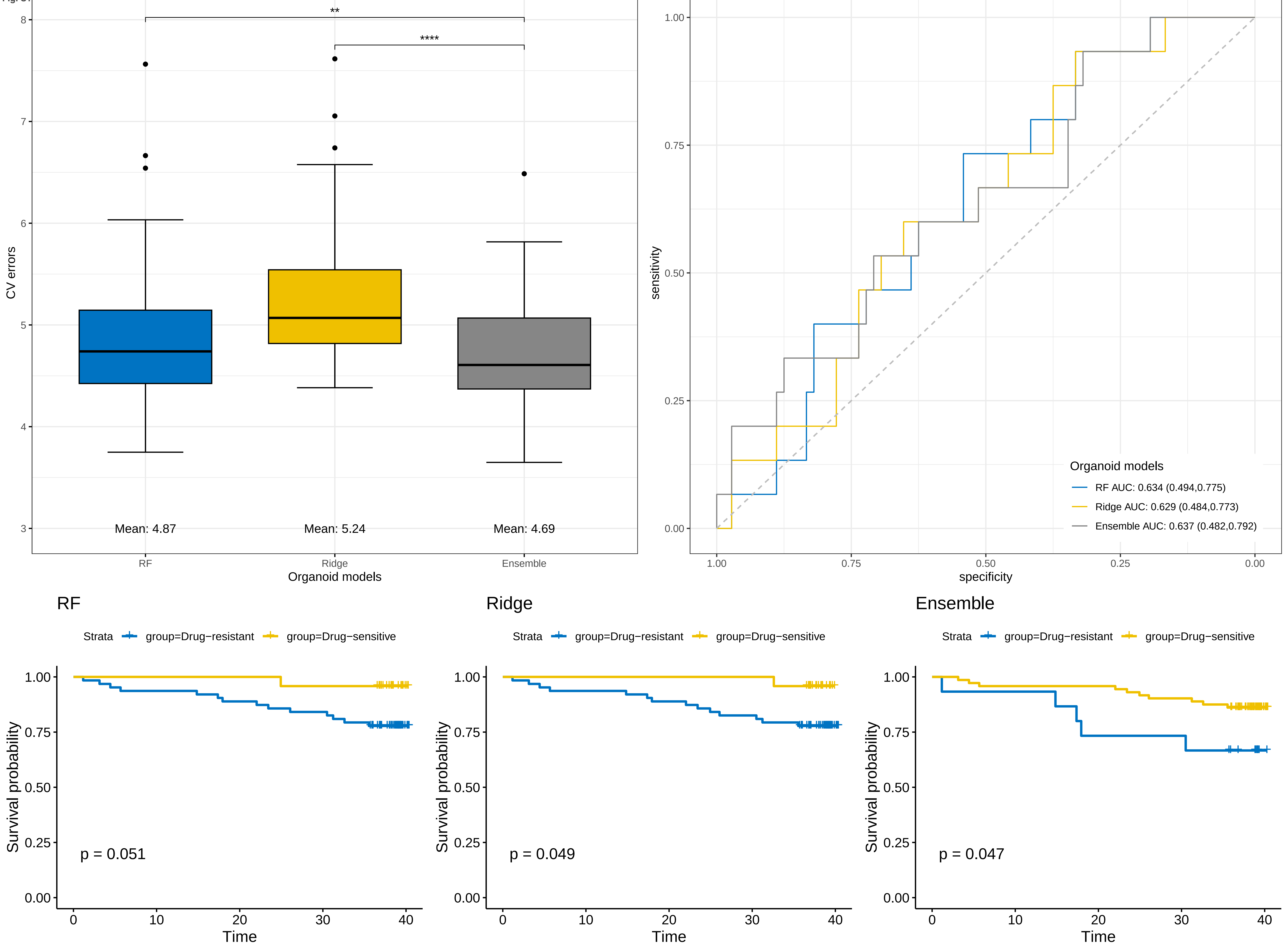

**
****
CV errors
Mean: 4.87
Mean: 5.24
Mean: 4.69
RF
Ridge
Ensemble
Organoid models
sensitivity
specificity
Organoid models
RF AUC: 0.634 (0.494,0.775)
Ridge AUC: 0.629 (0.484,0.773)
Ensemble AUC: 0.637 (0.482,0.792)
RF
Ridge
Ensemble
Strata
group=Drug−resistant
group=Drug−sensitive
Survival probability
Time
p = 0.051
p = 0.049
p = 0.047

Fig.S2

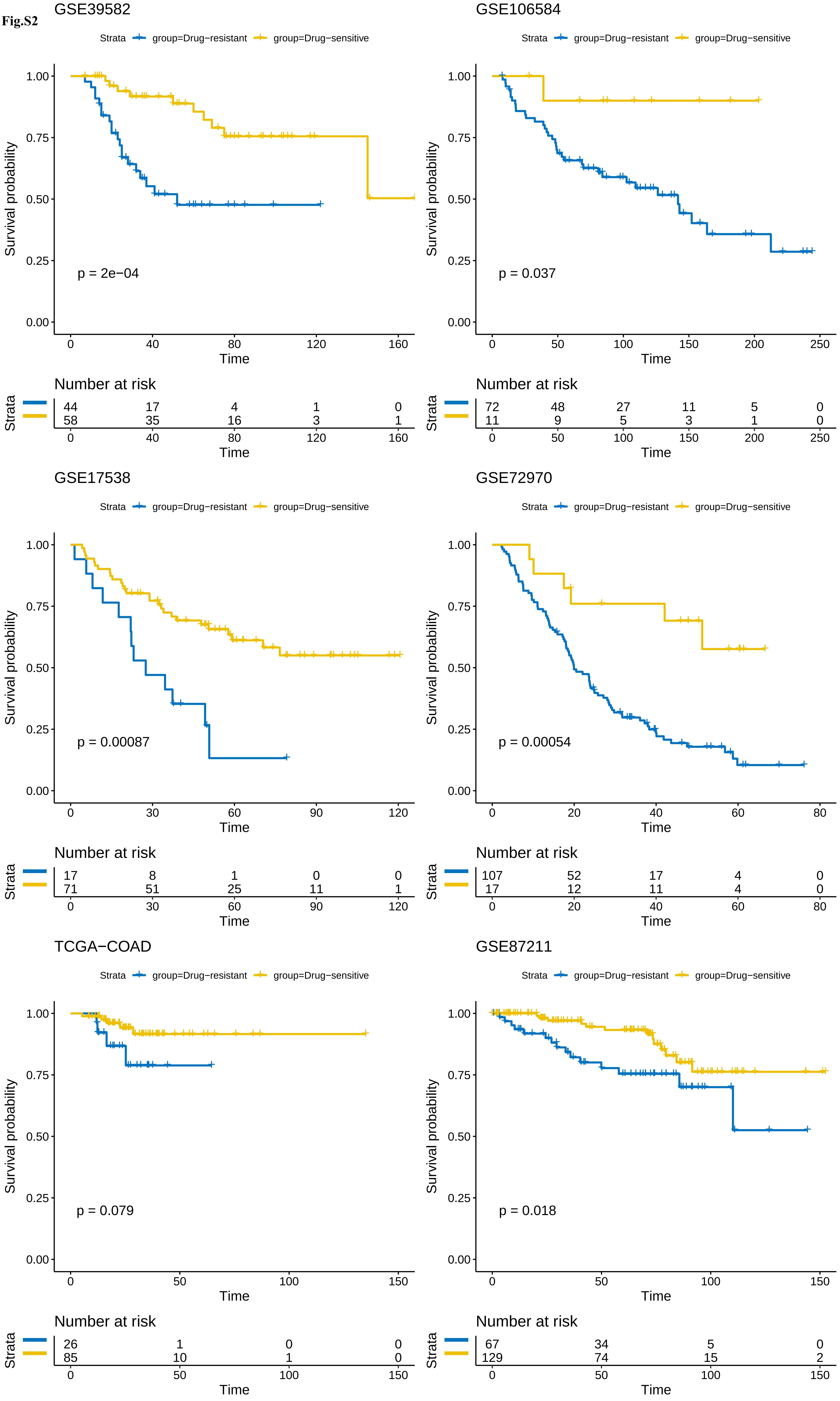
GSE39582
Strata group=Drug−resistant group=Drug−sensitive
Survival probability
p = 2e−04
Time
Number at risk
44 17 4 1 0
58 35 16 3 1
0 40 80 120 160
GSE106584
p = 0.037
72 48 27 11 5 0
11 9 5 3 1 0
0 50 100 150 200 250
GSE17538
p = 0.00087
17 8 1 0 0
71 51 25 11 1
0 30 60 90 120
GSE72970
p = 0.00054
107 52 17 4 0
17 12 11 4 0
0 20 40 60 80
TCGA−COAD
p = 0.079
26 1 0 0
85 10 1 0
0 50 100 150
GSE87211
p = 0.018
67 34 5 0
129 74 15 2
0 50 100 150

Fig.S3

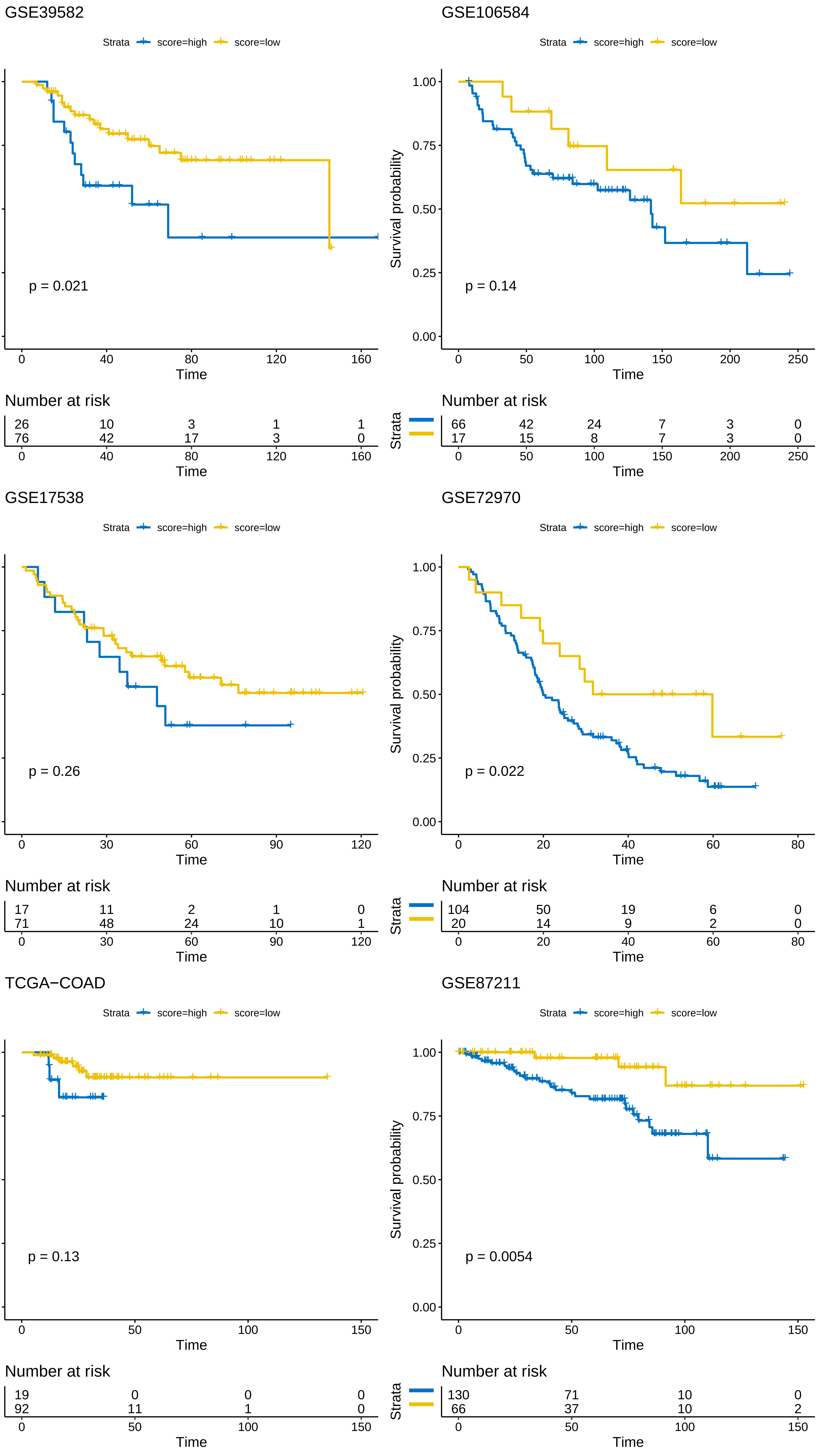
GSE39582
Strata score=high score=low
Survival probability
1.00
0.75
0.50
0.25
0.00
p = 0.021
0 40 80 120 160
Time
Number at risk
Strata
26 10 3 1 1
76 42 17 3 0
0 40 80 120 160
Time
GSE106584
Strata score=high score=low
Survival probability
p = 0.14
0 50 100 150 200 250
Time
Number at risk
Strata
66 42 24 7 3 0
17 15 8 7 3 0
0 50 100 150 200 250
Time
GSE17538
Strata score=high score=low
Survival probability
p = 0.26
0 30 60 90 120
Time
Number at risk
Strata
17 11 2 1 0
71 48 24 10 1
0 30 60 90 120
Time
GSE72970
Strata score=high score=low
Survival probability
p = 0.022
0 20 40 60 80
Time
Number at risk
Strata
104 50 19 6 0
20 14 9 2 0
0 20 40 60 80
Time
TCGA−COAD
Strata score=high score=low
Survival probability
p = 0.13
0 50 100 150
Time
Number at risk
Strata
19 0 0 0
92 11 1 0
0 50 100 150
Time
GSE87211
Strata score=high score=low
Survival probability
p = 0.0054
0 50 100 150
Time
Number at risk
Strata
130 71 10 0
66 37 10 2
0 50 100 150
Time

Fig.S4

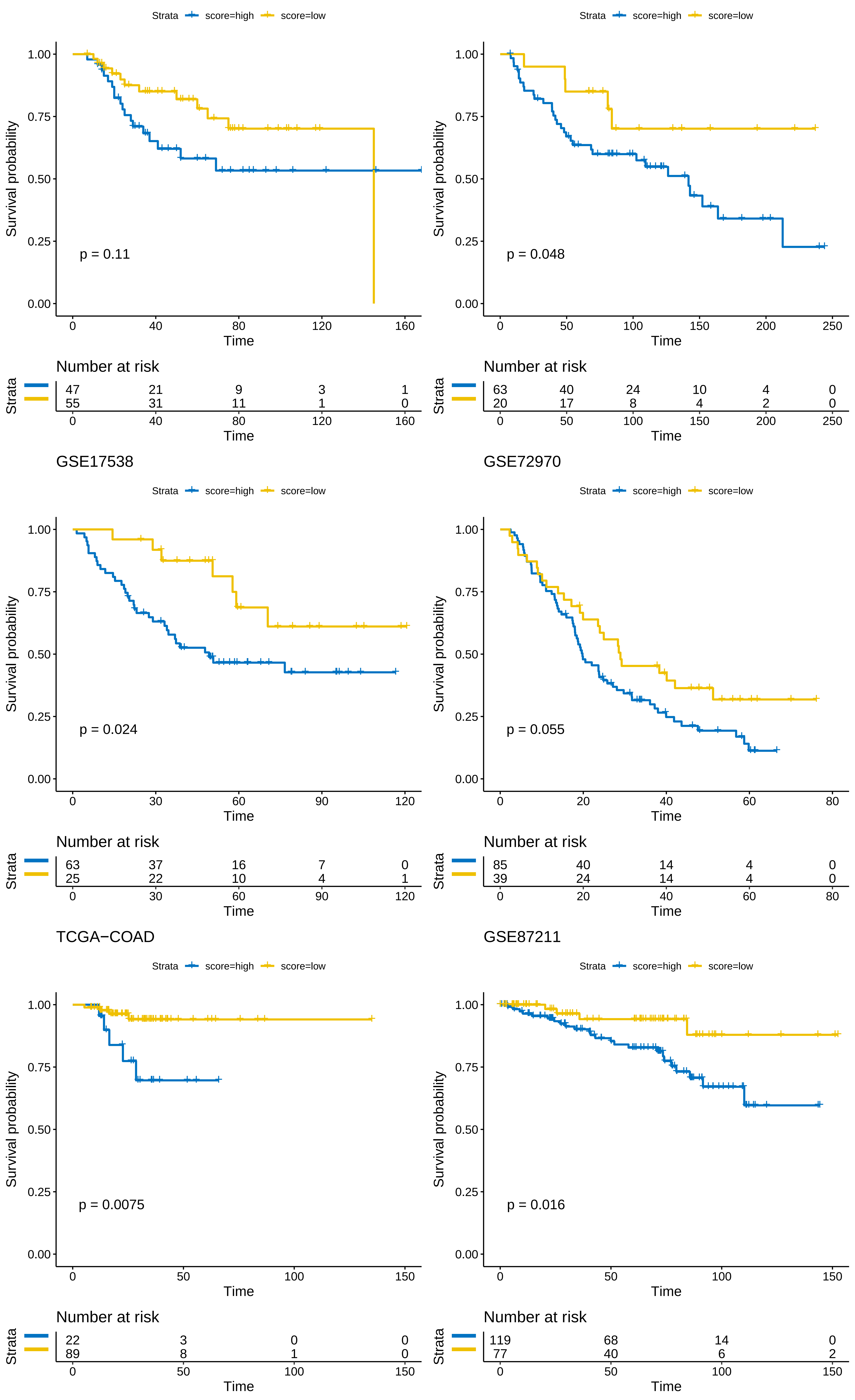
GSE39582
Strata score=high score=low
Survival probability
1.00
0.75
0.50
0.25
0.00
p = 0.11
0 40 80 120 160
Time
Number at risk
Strata
47 21 9 3 1
55 31 11 1 0
0 40 80 120 160
Time
GSE106584
Strata score=high score=low
Survival probability
p = 0.048
0 50 100 150 200 250
Time
Number at risk
Strata
63 40 24 10 4 0
20 17 8 4 2 0
0 50 100 150 200 250
Time
GSE17538
Strata score=high score=low
Survival probability
p = 0.024
0 30 60 90 120
Time
Number at risk
Strata
63 37 16 7 0
25 22 10 4 1
0 30 60 90 120
Time
GSE72970
Strata score=high score=low
Survival probability
p = 0.055
0 20 40 60 80
Time
Number at risk
Strata
85 40 14 4 0
39 24 14 4 0
0 20 40 60 80
Time
TCGA−COAD
Strata score=high score=low
Survival probability
p = 0.0075
0 50 100 150
Time
Number at risk
Strata
22 3 0 0
89 8 1 0
0 50 100 150
Time
GSE87211
Strata score=high score=low
Survival probability
p = 0.016
0 50 100 150
Time
Number at risk
Strata
119 68 14 0
77 40 6 2
0 50 100 150
Time

Fig.S5

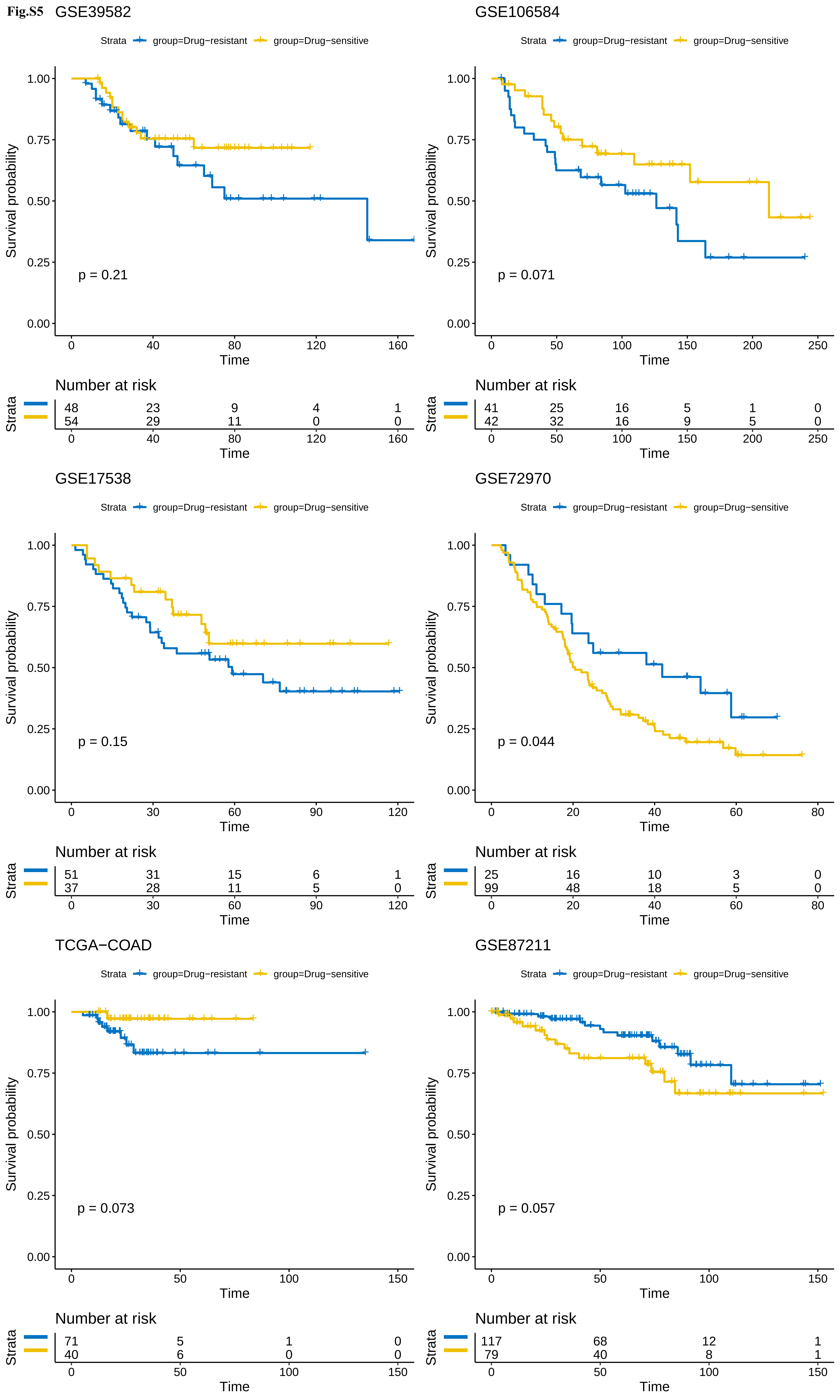
GSE39582
Strata group=Drug−resistant group=Drug−sensitive
Survival probability
1.00
0.75
0.50
0.25
0.00
p = 0.21
0 40 80 120 160
Time
Number at risk
Strata
48 23 9 4 1
54 29 11 0 0
0 40 80 120 160
Time
GSE106584
Strata group=Drug−resistant group=Drug−sensitive
Survival probability
1.00
0.75
0.50
0.25
0.00
p = 0.071
0 50 100 150 200 250
Time
Number at risk
Strata
41 25 16 5 1 0
42 32 16 9 5 0
0 50 100 150 200 250
Time
GSE17538
Strata group=Drug−resistant group=Drug−sensitive
Survival probability
1.00
0.75
0.50
0.25
0.00
p = 0.15
0 30 60 90 120
Time
Number at risk
Strata
51 31 15 6 1
37 28 11 5 0
0 30 60 90 120
Time
GSE72970
Strata group=Drug−resistant group=Drug−sensitive
Survival probability
1.00
0.75
0.50
0.25
0.00
p = 0.044
0 20 40 60 80
Time
Number at risk
Strata
25 16 10 3 0
99 48 18 5 0
0 20 40 60 80
Time
TCGA−COAD
Strata group=Drug−resistant group=Drug−sensitive
Survival probability
1.00
0.75
0.50
0.25
0.00
p = 0.073
0 50 100 150
Time
Number at risk
Strata
71 5 1 0
40 6 0 0
0 50 100 150
Time
GSE87211
Strata group=Drug−resistant group=Drug−sensitive
Survival probability
1.00
0.75
0.50
0.25
0.00
p = 0.057
0 50 100 150
Time
Number at risk
Strata
117 68 12 1
79 40 8 1
0 50 100 150
Time

Fig.S6

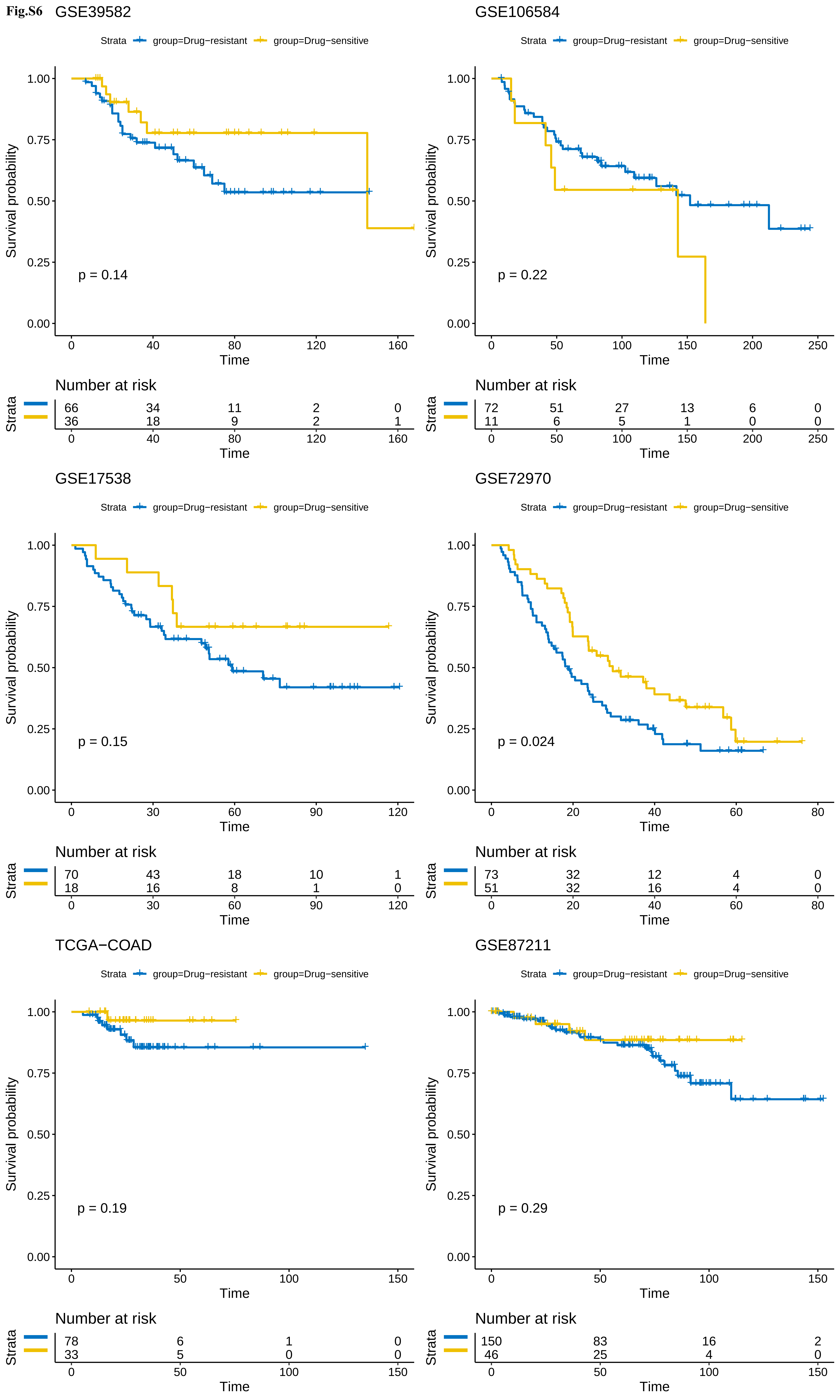
GSE39582
Strata group=Drug−resistant group=Drug−sensitive
Survival probability
1.00
0.75
0.50
0.25
0.00
p = 0.14
0 40 80 120 160
Time
Number at risk
Strata
66 34 11 2 0
36 18 9 2 1
GSE106584
p = 0.22
0 50 100 150 200 250
72 51 27 13 6 0
11 6 5 1 0 0
GSE17538
p = 0.15
0 30 60 90 120
70 43 18 10 1
18 16 8 1 0
GSE72970
p = 0.024
0 20 40 60 80
73 32 12 4 0
51 32 16 4 0
TCGA−COAD
p = 0.19
0 50 100 150
78 6 1 0
33 5 0 0
GSE87211
p = 0.29
0 50 100 150
150 83 16 2
46 25 4 0

Fig.S7

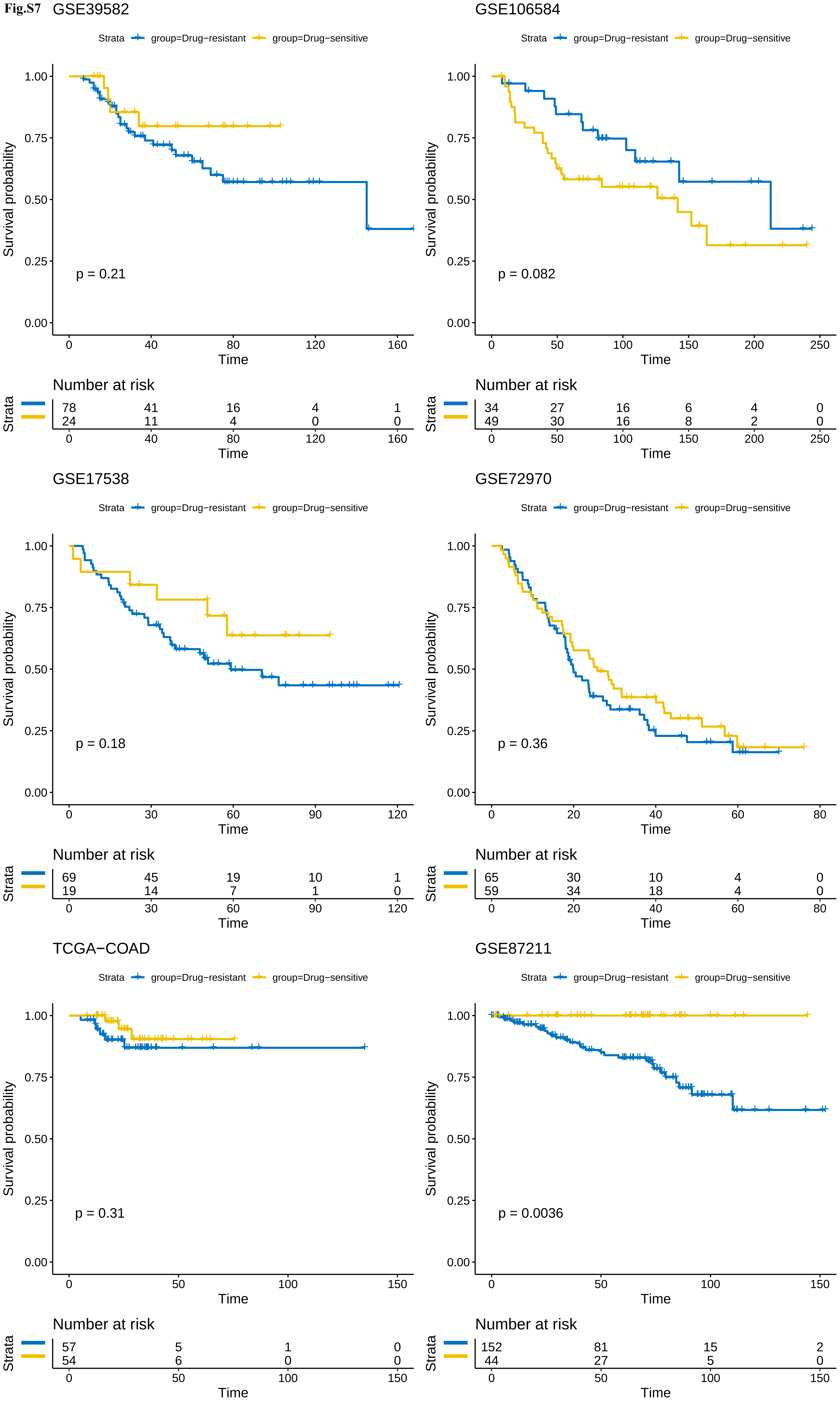

GSE39582
Strata group=Drug−resistant group=Drug−sensitive
Survival probability
1.00
0.75
0.50
0.25
0.00
p = 0.21
0 40 80 120 160
Time
Number at risk
Strata
78 41 16 4 1
24 11 4 0 0
0 40 80 120 160
Time
GSE106584
Strata group=Drug−resistant group=Drug−sensitive
Survival probability
1.00
0.75
0.50
0.25
0.00
p = 0.082
0 50 100 150 200 250
Time
Number at risk
Strata
34 27 16 6 4 0
49 30 16 8 2 0
0 50 100 150 200 250
Time
GSE17538
Strata group=Drug−resistant group=Drug−sensitive
Survival probability
1.00
0.75
0.50
0.25
0.00
p = 0.18
0 30 60 90 120
Time
Number at risk
Strata
69 45 19 10 1
19 14 7 1 0
0 30 60 90 120
Time
GSE72970
Strata group=Drug−resistant group=Drug−sensitive
Survival probability
1.00
0.75
0.50
0.25
0.00
p = 0.36
0 20 40 60 80
Time
Number at risk
Strata
65 30 10 4 0
59 34 18 4 0
0 20 40 60 80
Time
TCGA−COAD
Strata group=Drug−resistant group=Drug−sensitive
Survival probability
1.00
0.75
0.50
0.25
0.00
p = 0.31
0 50 100 150
Time
Number at risk
Strata
57 5 1 0
54 6 0 0
0 50 100 150
Time
GSE87211
Strata group=Drug−resistant group=Drug−sensitive
Survival probability
1.00
0.75
0.50
0.25
0.00
p = 0.0036
0 50 100 150
Time
Number at risk
Strata
152 81 15 2
44 27 5 0
0 50 100 150
Time
